# Head Trauma Exposure in Mixed Martial Arts

**DOI:** 10.3390/ijerph192013050

**Published:** 2022-10-11

**Authors:** Katarzyna Mańka-Malara, Elżbieta Mierzwińska-Nastalska

**Affiliations:** Department of Prosthodontics, Medical University of Warsaw (Poland), 02-097 Warsaw, Poland

**Keywords:** combat sports, martial arts, trauma, head injury, mouthguards, injury prevention, concussion, sport performance

## Abstract

Combat sports training involves a high risk of head injury. Previously published research on head trauma exposure in MMA evaluated only the knockouts (KO), without calculating all head strikes. The aim of the research was to evaluate the total head trauma exposure during MMA competitions among male and female fighters. Two thousand four hundred and eighty-eight MMA fights from all numbered UFC events between 2000 and 2021 were analyzed. A database containing the results from officially published scorecards with information such as the outcome of a fight, its duration, number of strikes (significant and total amount of hits) depending on location and knockdowns was created. Additional video verification of the knockout technique was carried out. The athletes received an average of 2.41 significant head strikes out of a total of 6.30 head strikes per minute. Head strikes were more common in female fights than in male. Women executed more total and significant head strikes per minute than men. Head trauma caused the ending of 31.6% of all fights—more often in male fights (32.2%) than female (23.1%). It was the most common cause of knockouts—88.1%. Professional fights in mixed martial arts involve high exposure to head trauma. A careful evaluation of the risk involved in training in such a discipline is necessary to provide adequate prevention methods.

## 1. Introduction

Mixed martial arts (MMA) requires versatile skills as the fight contains techniques from various combat sports. A combination of grappling and striking skills must be supported by specific physiological characteristics to achieve top results [1]. This fight style is perceived as extremely violent and dangerous. Its popularity is increasing despite some medical associations recommending that, due to the high injury risk, MMA and boxing should be banned [2,3,4]. Adequate prevention of injuries in MMA is indispensable—in the United States alone, 5.5 million adolescents and 3.2 million children, younger than 13 years of age, train in this sport—an amount comparable to those training in football or baseball [5]. A systematic meta-analysis of injuries in MMA conducted in 2014 reported 22.9 injuries for every 100 athlete exposures [6]. They were most common in the head and face region (66.8–78%) [7]. Fares et al. [8] described a total of 288 head injuries in 408 fights, with the highest incidence of traumatic brain injury (TBI). Retrospective analysis showed that in MMA head trauma is a serious problem—repeated mild traumatic brain injury may cause chronic traumatic encephalopathy and impaired cognitive functions [9]. Curran-Sills et al. [10] recommended that there should be guidelines created regarding pre- and post-bout neuroimaging, standard medical screening introduced, the development of competent ringside physician groups, and active oversight by the Combative Sports Commission during the matchmaking process. There have been associations made between head injuries in MMA and potential risk factors such as age, sex, weight, match outcome and bout length. The meta-analysis showed that contests in the heavyweight class title fights, longer bouts, fights ended due to KO/TKO (knockout/technical knockout), and fight losing was associated with higher rates of injuries [11].

Previously published research on head trauma exposure in MMA evaluated only the knockouts (KO), without calculating all head strikes that a fighter receives during a bout. The analysis of total exposure is very important. Head impact frequency including impact magnitude and the time interval between impacts are characteristics associated with chronic neurological injuries and have been previously used to quantify brain trauma exposure in sport [12,13]. Repeated mild traumatic brain injury causes chronic traumatic encephalopathy and impaired cognitive functions. Research shows that head impacts can lead to negative neurophysiological consequences and the onset of chronic brain injury regardless of the presence of concussive signs. Athletes may be diagnosed with chronic traumatic encephalopathy with no history of concussion, suggesting that even sub-concussive hits are sufficient to lead to the development of degenerative brain disease [14,15,16]. Additionally, strikes that do not result in concussion may still cause trauma to teeth, bones, temporomandibular joints, and soft tissues while professional fighters in MMA use only mouthguards as protection in the head area which provides only small absorption of impact forces [17,18,19]. Another important thing is that previously published studies included only male fighters. The first female fight in the Ultimate Fighting Championship (UFC)—probably the most well-known MMA organization—took place on 23 February 2013. Despite years passing, there is still a gap of knowledge concerning the differences in head trauma exposure, and performance between sexes in mixed martial arts. The presented research evaluated the head trauma exposure in mixed martial arts during sports competitions and compared the results between male and female fighters.

## 2. Materials and Methods

### 2.1. Data Source and Description

**Figure 1:**
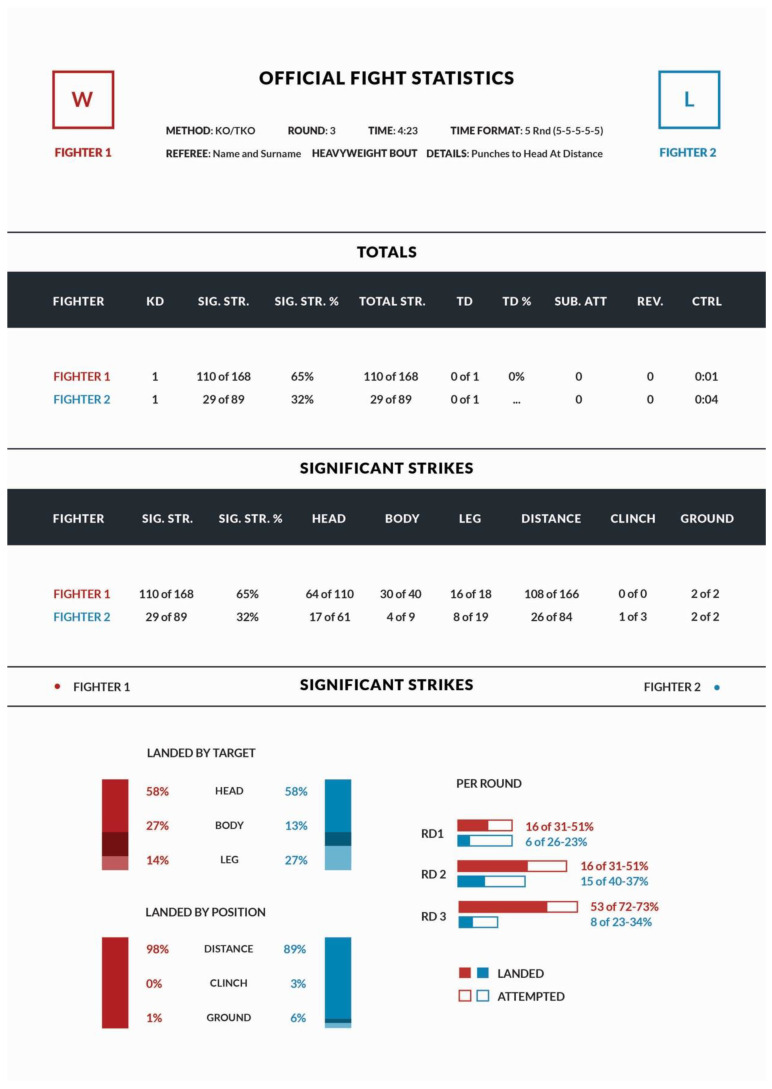
Schematic view of fight statistics used for database.

Two thousand, four hundred and eighty-eight (169 female and 2319 male) MMA fights from all numbered Ultimate Fighting Championship (UFC) events between 2000 and 2021—from 28 “High Stakes” (the beginning of unified rules) to 265 “Lewis vs. Gane”—were analyzed. To maintain the same level of proficiency of male and female athletes, only fights from numbered events, where top fighters from all divisions compete, were included. Only 24 overturned fights, having status “no-contest” due to failed drug test or illegal technique were not included in the analysis. Official fight statistics, published by the UFC on the site ufcstats.com were used. Fight evaluation is made by professional referees and the official number of strikes is calculated by the UFC team and published online. Such results are used by this organization to calculate rankings and compare the fighter’s effectiveness. A schematic view of fight results which were manually rewritten into a database is shown in Figure 1. Each result of the fight was coded as KO/TKO—knockout/technical knockout, SUB—submission, unanimous decision, majority decision, split decision, DQ—disqualification or overturned. The Round number means in which round the fight ended. Time format shows how many rounds were planned for the fight—for instance, most title fights are scheduled for five rounds which would be written as 5 Rnd (5-5-5-5-5). Details provided for each KO/TKO fight were used to determine the cause of the knockout. For fights where the ending technique was in the head (such as punch to head at a distance or punches to head on the ground), the knockout was calculated in the subsequent analysis as connected with head trauma. If a description was not clear, and the cause of ending could not be clearly stated—for instance it was described as elbows to head from side control submission—the video of the fight was searched and based on the analysis of publicly available video, it was classified whether it could be stated that the knockout was due to head trauma. Head trauma was noted only when an impact resulted in visible motion of the head, the impact type was visible on the video, and the moment of impact could be identified [12,13]. The used abbreviations in the official UFC fight statistics are KD—knockdowns, SIG. STR.—significant strikes, SIG. STR.%—significant strikes percentage, TOTAL STR.—total strikes, TD—takedowns, TD%—takedowns percentage, SUB. ATT.—submission attempts and CTRL.—control time. Significant strikes include every strike at distance and power strikes in the clinch and on the ground. Total strikes included also small, short strikes in the clinch and on the ground.

### 2.2. Statistical Analysis

All statistical analyses were performed with the use of SPSS 27 software (version 28, SPSS Inc., Chicago, IL, USA), and all the graphs were prepared with the use of Microsoft Excel. There were two main types of analyses: (1) comparison of the dependent variables (such as number of head strikes per minute, number of knockdowns per fight, and duration of fight) between female and male fights, and (2) comparisons of the same dependent variables across the weight categories. The comparisons between female and male fights were either performed for the whole dataset or limited to equivalent weight categories.

As the database included many more male than female fights, and the number of fights included in the analyses varied across the weight categories, we employed non-parametric methods of data analyses. Therefore, Mann–Whitney tests were used for comparisons of interval and ratio variables between female and male fights and Kruskal–Wallis tests were applied for comparisons across the weight categories. If the dependent variables were nominal, Chi-square tests we applied.

The analyses were organized according to the dependent variables. First, we compared the number of head strikes per minute between female and male fights (for the complete dataset), across the weight categories, separately for female and male fights, and between female and male fights within equivalent weight categories. Second, we compared the number of knockdowns between female and male fights, and across the weight categories. Additionally, we compared the number of knockdowns between female and male fights in corresponding weight categories. Third, we compared the numbers of all strikes between female and male fights in equivalent weight categories. Next, we compared the distribution of fight results between female and male fights and across weight categories. Finally, we analyzed fight durations in accordance with fighters’ gender, weight category, and the result of the fight. In all analyses, we considered *p*-values < 0.05 as significant, and in cases of multiple comparisons (post hoc comparisons in Kruskal–Wallis tests) we applied a Bonferroni correction for multiple comparisons.

## 3. Results

There were 4976 fighters—338 females and 4638 male—included in the evaluation. Fighters received between 0 and 51 significant strikes to the head per minute of combat (mean: 2.41; median: 1.67). 

### 3.1. Head Strikes

**Figure 2:**
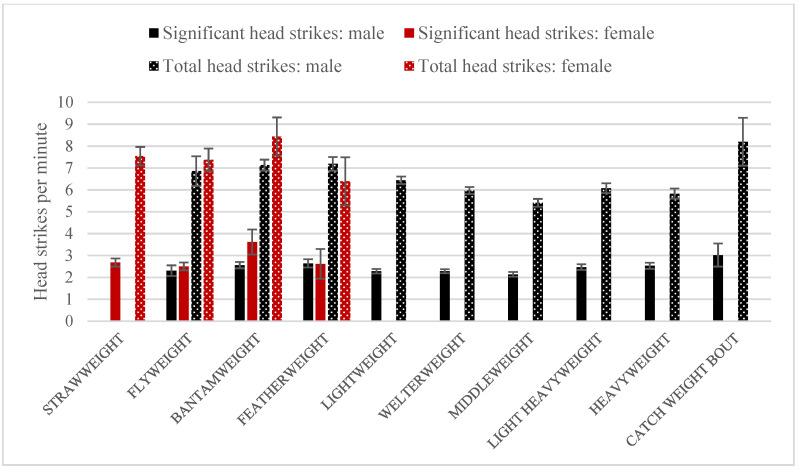
The number of significant (filled) and total (dotted) head strikes per minute in male (black) and female (red) fights. The error bars present standard errors.

Head strikes were more frequently delivered by female than male fighters (Figure 2). Females executed more total head strikes per minute than males (females: M = 7.73, SD = 6.63; males: M = 6.20, SD = 5.34; Mann–Whitney U = 920,094, *p* < 0.001) and more significant strikes per minute (females: M = 2.95, SD = 3.94; men: M = 2.37, SD = 3.06; Mann–Whitney U = 919,731.5, *p* < 0.001). In male fights, both the number of total (*H(12) =* 146.2, *p* < 0.001). and the significant (*H(12) =* 163.5, *p* < 0.001) head punches per minute differed between the weight categories. In particular, the number of head punches per minute was higher in the lower (bantamweight and featherweight) than in the higher weight categories (lightweight to middleweight) as indicated by post hoc tests (all corrected *p*-values < 0.05). In women’s bouts, there were no differences between weight categories in the number of total or significant head strikes in the women’s bouts (both corrected *p*-values > 0.05).

We also compared the results between equivalent weight categories. There were statistically significant differences in the number of total and significant strikes to the head per minute of the fight. In the flyweight division, women delivered both more significant (Mann–Whitney’s U = 6,301,000, *p* = 0.010), and more total strikes (Mann–Whitney’s U = 6,124,000, *p* = 0.030). In the bantamweight, no significant differences were found in either the number of significant (*p* > 0.05) or the number of total strikes (*p* > 0.05). Furthermore, no statistically significant differences were found in the featherweight in either the number of significant (*p* > 0.05) or the number of total punches (*p* > 0.05).

### 3.2. Knockdowns

**Figure 3:**
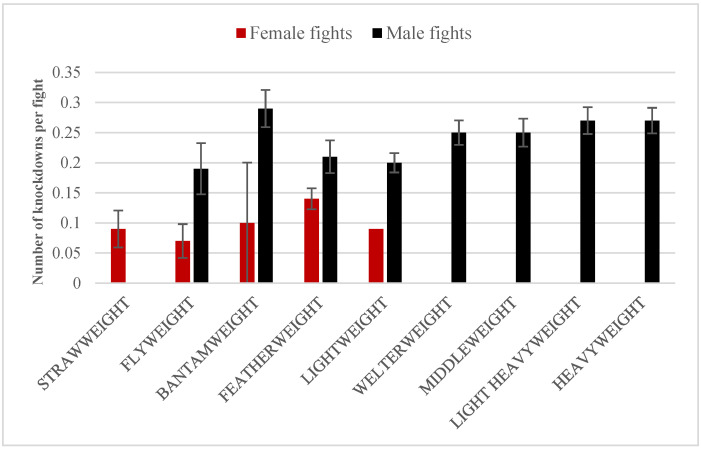
Number of knockdowns per fight in female (red) and male (black) fights. The error bars present standard errors.

The number of knockdowns (KD) per fight is presented on Figure 3. Using the Kruskal–Wallis test, it was verified whether there were differences in the frequency of KDs across weight categories (separately for men’s and women’s bouts). A significant effect of category was found in male fights (U = 21.76, *p* = 0.005). The only statistically significant difference was found for the lightweight-heavyweight pair (adjusted *p* = 0.014). 

### 3.3. All Strikes

**Figure 4:**
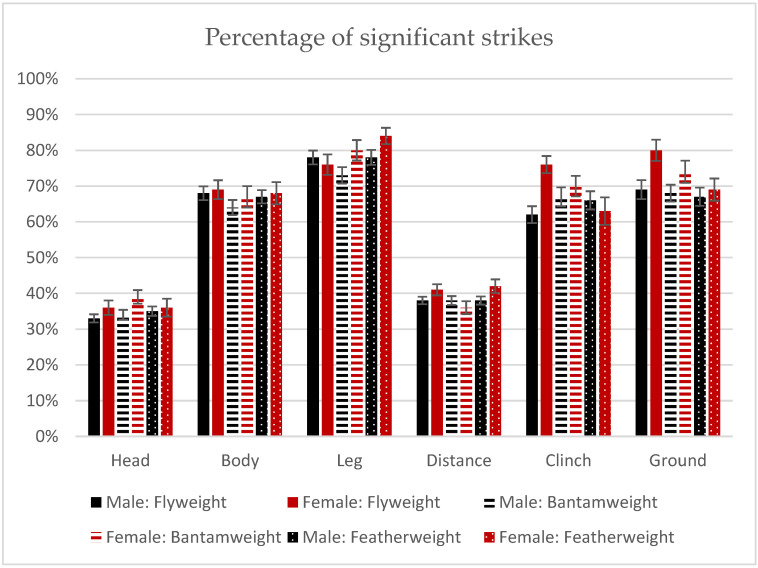
Percentage of significant strikes in men’s (black) and women’s (red) fights in flyweight (filled), bantamweight (striped) and featherweight (dotted) categories.

The percentages of all significant strikes derived by males and females in the corresponding weight categories were also compared (Figure 4). In the flyweight division, no sex differences were found for significant strikes in the head, body, and leg (all *p* > 0.05). Statistically significant differences were found for clinch (U = 4559.5, *p* < 0.001), distance (U = 6115.5, *p* = 0.031) and ground (U = 2725, *p* = 0.039) strikes. In all these types, females had higher percentages of significant punches. In the bantamweight category, there were no statistically significant gender differences for body, distance, and clinch (all *p* > 0.05). Statistically significant differences were found for significant strikes (U = 22695.5, *p* = 0.005), head punches (U = 5685, *p* = 0.004), leg (U = 4009.5, *p* = 0.027) and those performed while fighting on the ground (U = 2725, *p* = 0.034). Again, in all these types of strikes, the percentages in women’s fights were higher than in men’s fights. No statistically significant differences were found in the featherweight category.

### 3.4. Fight Results

Fight results were statistically significantly different between male and female. Women’s bouts were relatively more likely to end due to split decision (14.8% vs. 9.1% in men) and unanimous decision (41.4% in women; 35.6% in men), and relatively less likely to end due to KO/TKO (knockout/technical knockout) (26.0% of women’s bouts and 36.5% of men’s bouts). Among all fights, 31.6% ended due to head injury (Table 1). It was more often the cause of fight ending in male (32.2%) than female fights (23.1%; Chi2(1) = 6.03, *p* < 0.001). The percentage of fights ended by KO due to head injury differed significantly between weight categories (Table 2; Chi2(12) = 109.24, *p* < 0.001). Relatively, fights were most often ended by KO due to head injury in the heaviest categories—heavyweight (54% of fights) and light heavyweight (38% of fights)—and least often in the lightest flyweight category (12.5% of fights). Among all fights (N = 2488), 855 fights that ended by KO were identified. Within these fights, fights ended due to head injury accounted for 88.1% of fights (780 fights out of 855). The percentage of fights terminated by KO due to head injury among all fights terminated by KO did not differ between male and female fights (Table 3; Chi2(1) = 0.01, *p* = 0.916). Additionally, the percentage of fights terminated by KO with a head injury did not differ between title fights and other fights (Chi2(1) = 0.33, *p* = 0.566).

### 3.5. Fight Duration and Result

The fight duration differed significantly between female (M = 12 min 5 s; SD = 6 min 16 s) and male fights (M = 10 min 27 s; SD = 6 min 11 s; Mann–Whitney U = 225,230, *p* < 0.001). Female bouts were on average one and a half minutes longer. Because our database included relatively more women’s title fights than men’s, we verified whether the differences in fight duration were related to this unequal proportion. We tested the times of title and other fights and compared them between sexes. The times of title bouts (N = 261) were not significantly different between female (n = 34; M = 13 min; SD = 10 min 24 s) and male fights (n = 227; M = 15 min 22 s; SD = 9 min 4 s; Mann–Whitney U = 3,272, *p* = 0.142). The times of the remaining fights (N = 2,488) differed significantly for women (n = 135; M = 11 min 51 s; SD = 4 min 44 s) and men (n = 2,092; M = 9 min 55 s; SD = 5 min 32 s; Mann–Whitney’s U = 170,502, *p* < 0.001). Female fights were on average two minutes longer. Because statistically significant differences in fight times between sexes occurred only in the times of non-title fights, which accounted for 89.5% of all analyzed, it was determined that the differences in fight times between men and women were not due to the title/non-title fight proportion. 

To assess at what minute of the fight the head injuries that ended the fight occurred, the duration of 785 fights ended by KO with a head injury in different weight categories were analyzed (Table 4). These fights lasted from 5 s to 24 min and 10 s. The average duration was 6 min and 6 s. The times of fights terminated by head injury were not significantly different between female (n = 39; M = 6 min 48 s; SD = 5 min 2 s) and male fights (n = 746; M = 5 min 55 s; SD = 4 min 29 s; Mann–Whitney U = 16,000, *p* =. 293). KO with head injury (ending the fight) occurred later in title fights (n = 103; M = 8 min 57 s; SD = 6 min 40 s) than in non-title fights (n = 682; M = 5 min 30 s; SD = 3 min 54 s; U Mann–Whitney = 45,238, *p* < 0.001). Due to the low number ended by this cause in the men’s catch weight bouts and flyweight categories and in all women’s categories, these fights were not included in the statistical analysis comparing the differences between weight categories. The Kruskal–Wallis test showed that the duration of the fight to termination by head injury differed significantly between weight divisions (*p* = 0.007). The only statistically significant difference was between the featherweight and light heavyweight categories (adjusted *p* = 0.012), i.e., fights in the light heavyweight category terminated by KO due to head injury were shorter than the corresponding fights in the featherweight category.

## 4. Discussion

The presented research shows that there is high exposure to head trauma in MMA. Fighters receive on average 2.41 significant head strikes per minute of a fight. That number is higher in female fights than male—women execute a median of 2.95 significant strikes per minute while men, 2.37. Significant strikes are those performed at a distance, power strikes in a clinch, or on the ground; thus we may assume that this represents strong impacts which a fighter receives to the head during combat. Full exposure—the median of all punches to the head, including small, short strikes in the clinch and on the ground—was 7.73 head strikes per minute in female fights and 6.2 in male fights. In MMA, various techniques from different combat sports are allowed. For instance, hammer strikes using a side of a hand to a lying opponent may cause significant damage—the head is lying on the ground, and the energy is absorbed by the skull. Short strikes in the clinch or on the ground are not calculated by UFC as significant—these are not performed from a distance—but their numbers are high as is shown in the high number of total head punches. Additionally, short repetitive punches often result in KO. Ninety per cent of TKOs are as a result of repetitive strikes [20]. Between the time of the strike causing KO and match stoppage the athlete receives on average 2.6 additional strikes. Further research should concentrate on this aspect as repeated mild traumatic brain injury may cause chronic traumatic encephalopathy and impaired cognitive functions [21,22]. The evaluation of the full number of head strikes during professional MMA fights has not been previously described in the literature. 

The occurrence of knockouts—strikes, kicks, or a combination of techniques that cause the inability to continue fighting and ends the combat—or technical knockouts, when a referee stops the contest when a competitor is unable to defend, was 26% in female fights and 36.5% in male. Of all fights, 31.6% ended due to a head injury. Hutchison et al. [20] calculated the occurrence of KO in UFC between 2006 to 2012 as 6.4 per 100 athlete exposures (12.7% matches). They used official scorecard analysis and video analysis of publicly available digital records. A total of 844 matches was included—433 undercard matches and 411 main-card matches. Data included only male fighters and the authors did not calculate the total number of head strikes. The lower percentage of knockouts in their study may be a result of the smaller number of evaluated fights or including undercard matches. Ngai et al. [23] provided the rate of 64.9 injuries in 1000 fight minutes and 15.4 severe concussions in every 1000 athletes’ expositions. They evaluated 635 professional male MMA fights. McCain et al. [24] included 54 female fights in the analysis of 711 amateur and professional MMA fights. Some 35.2% of female fights and 28.6% of male fights ended with KO/TKO. The decision ended 38.9% of female and 14% of male combats. Data regarding female boxing show that health problems caused by competitions are 0.30% per year—less frequent than in male boxing [24]. Authors state that this discipline should finally rid itself of the label “experimental sports” being as safe as male competitions. Davis et al. [25] evaluated the performance of female and male amateur boxers, showing that neither mass nor height influenced winning. A successful strategy for a women’s boxing match was maintaining a high activity rate and a lower ratio of total attack to landed punches. The number of punches to the head and body had a ratio of about 21 to 1—much higher than the 5:1 reported for men [25,26,27]. A higher number of head strikes made by women was also visible in our research—female fighters made more total and significant head punches than men. However, the different characteristics of boxing fights make those results difficult to compare. Jansen et al. [28] compared the number of head strikes between men and women in MMA training, stating that men experience a greater number of head impacts than women. However, they included only 4 women among the 23 participants, and data was collected from 53 amateur training episodes and 6 competitions.

The presented results showed that head trauma was a more common cause of knockout in males than females—it was the reason for ending 32.2% of male and 23.1% of female fights. In contrast, head strikes were more frequently delivered by females than males—professional female fighters in MMA make more head strikes than men—7.73 per minute of the fight. Reports suggest that in sports played with the same rules for males and females, the incidence rate of sport-related concussion is higher in female athletes [29,30,31]. Additionally, according to the literature, women may have a longer recovery time, and a higher risk of concussions in contact sports [32,33]. Surprisingly, females with a history of concussion demonstrate no significant effects on cerebral blood flow which may mean that the effects of concussion are intrinsically more heterogenous for female athletes or that women have greater variability in the neurobiological response to injury [34]. For instance, the vulnerability to concussion may depend on the phase of the menstrual cycle as higher levels of estrogen and progesterone confer a neuroprotective role following traumatic brain injury [35,36]. Additionally, females have lower head mass, and neck strength, are smaller, and prone to stretch injury axons in the central nervous system [37,38,39]. Jansen et al. [28] stated that there are no significant differences in terms of head impact magnitude between sexes in boxing and MMA. The sex of the fighter, however, moderates the relationship that the number of professional fights has with cognition and brain volumes [40]. Subcortical smaller volumes were associated with a greater number of professional fights and that relationship was much steeper for men. Additionally, a greater number of professional fights was associated with poorer verbal performance among male fighters, while there was an inverse relationship for women. Although there is evidence of sex differences in the clinical presentation of concussion, it is currently unclear whether they are associated with underlying differences in concussion neurobiology between men and women [41,42,43]. Sex-based differences in reaction to head trauma in mixed martial arts must be considered. 

The major limitation of the presented report concerning head trauma is the methodology based on officially reported fights statistics. The lack of clinical examination influences the possibility to classify a type of head injury and its severity. The observational characteristic of this research means that we could report only the incidences of knockouts but could not precisely state the number of concussions. Additionally, when reporting the number of strikes, we could not evaluate whether they resulted in head trauma but only evaluate the possibility of it. The calculation of punches and fight statistics was carried out by the UFC organization; therefore, it is difficult to verify its accuracy. However, we may assume that the calculation was carried out by experts, as those are official fight results. The additional video verification, applied where the details of the fight outcome were unclear, was a subjective decision, based on the visible motion of the head, the impact type, and the possibility of identifying the moment of impact. This classification could influence the results obtained. In this research, we decided to include only the numbered UFC events—this means that we had a high percentage of title fights and only top fighters included in the statistics. Such an evaluation performed for undercard fights or amateur MMA competitors could give other results, as the fighter’s experience and proficiency may influence fight results, the frequency of knockouts, or the number of strikes.

Mixed martial arts is a popular, but dangerous discipline. We believe that due to high exposure to head trauma, training in this discipline is a health hazard. The exposure to head trauma calculated in the current research may be used to evaluate the recommended frequency of health monitoring of professional fighters. The high number of total head punches should be considered when planning injury prevention, as head trauma is the main cause of knockouts, and a fighter receives many punches to the head. It would be recommended to introduce safeguards or procedures to reduce the risk of head injuries. The professional youth competitions should not be organized as participation in professional MMA fight means the possibility of brain damage due to a high number of head strikes. The differences between male and female fights should be considered when planning training strategies and injury prevention.

## 5. Conclusions

Professional fights in mixed martial arts involve high exposure to head trauma. During combat, a fighter receives 2.41 significant head strikes per minute on average—2.95 ± 3.94 in female fights and 2.37 ± 3.06 in male fights. There were also 7.73 ± 6.63 total head strikes in female and 6.2 ± 5.34 in male fights. The number of head punches (total and significant) in male fights was higher in the lower weight categories—such as bantamweight and featherweight. In women’s fights, such differences between divisions were not found. When comparing equivalent male and female divisions, there were statistically significant differences in total and head strikes (more performed by women) in the featherweight category. For all types of strikes, females had more significant punches in clinch, distance and on the ground in the male and female flyweight divisions, and in body strikes and those in distance and clinch in the male and female bantamweight divisions.

Women’s fights were on average one and a half minutes longer. Over thirty percent of all fights (31.6%) ended due to knockout with a head injury (32.2% of female and 23.1% of male fights). Most often, it was a cause of fight ending in the heaviest divisions—54% of heavyweight and 38% of light heavyweight fights. Head trauma was the most common cause of knockouts—it accounted for 88.1% of fights ended with KO. Careful evaluation of the risk involved in training for such a discipline is necessary to provide adequate prevention methods. 

## Figures and Tables

**Table 1:** Fights ended due to knockout (KO) with head injury by fighter gender.

Fight Result	M	W	Total
KO with head trauma	32.2% (746)	23.1% (39)	31.6% (785)
Other cause	67.8% (1573)	76.9% (130)	68.4% (1703)
**Total**	**2319**	**169**	**2488**

**Table 2:** Fights ended due to the KO with head injury versus all fights divided by weight category. The percentages and (raw numbers) are presented.

	Weight Category	KO with Head Trauma	Other Reason	Total
**Male** **fights**	Flyweight	12.5% (9)	87.5% (63)	72
	Bantamweight	28.7% (49)	71.3% (122)	171
	Featherweight	26.9% (49)	73.1% (133)	182
	Lightweight	24.5% (108)	75.5% (333)	441
	Middleweight	31% (111)	69% (247)	358
	Welterweight	29.5% (143)	341 (70.5%)	484
	Light heavyweight	38% (116)	62% (189)	305
	Heavyweight	54% (156)	46% (133)	289
	Catch weight bout	29.4% (5)	70.6% (12)	17
**Female** **fights**	Strawweight	16.9% (11)	83.1% (54)	65
	Flyweight	16.7% (6)	83.3% (30)	36
	Bantamweight	31.6% (18)	68.4% (39)	57
	Featherweight	36.4% (4)	63.6% (7)	11
**Total**		**31.6% (785)**	**68.4% (1703)**	**2488**

**Table 3:** Fights ended due to the KO with head injury versus all fights divided by male and female fights.

Fight Result	M	W	Total
KO with head trauma	88.1% (741)	88.6% (39)	88.1% (780)
Other reason	11.9% (100)	11.4% (5)	11.9% (105)
**Total**	**841**	**44**	**885**

**Table 4:** The duration of a fight ended by KO with a head injury depending on the weight category.

	Weight Category	N	Duration of Fight to End by KO with Head Injury	
			M	SD
**Male fights**	Flyweight	9 *	4 min 30 s	1 min 33 s
	Bantamweight	49	7 min 36 s	6 min 3 s
	Featherweight	49	7 min 36 s	4 min 35 s
	Lightweight	108	5 min 41 s	4 min 20 s
	Middleweight	111	6 min 8 s	3 min 59 s
	Welterweight	143	5 min 37 s	4 min 37 s
	Light heavyweight	116	4 min 58 s	3 min 41 s
	Heavyweight	156	5 min 59 s	4 min 40 s
	Catch weight bout	5 *	3 min 24 s	1 min 26 s
**Female fights**	Strawweight	11 *	7 min 12 s	4 min 45 s
	Flyweight	6 *	8 min 54 s	1 min 55 s
	Bantamweight	18 *	6 min 25 s	5 min 50 s
	Featherweight	4 *	4 min 18 s	5 min 13 s
**Total**		**785**	**5 min 57 s**	**4 min 31 s**

* Marked weight categories not included in the comparison of the duration of fights ended by KO with head injury.

## Data Availability

Statistics used for this study are available online: ufcstats.com. Evaluated data are available on reasonable request form the authors of this article.
